# Natural history bycatch: a pipeline for identifying metagenomic sequences in RADseq data

**DOI:** 10.7717/peerj.4662

**Published:** 2018-04-16

**Authors:** Iris Holmes, Alison R. Davis Rabosky

**Affiliations:** Department of Ecology and Evolutionary Biology, University of Michigan Museum of Zoology, University of Michigan, Ann Arbor, MI, USA

**Keywords:** Metagenomics, DNA barcoding, Parasitology, Museum resources

## Abstract

**Background:**

Reduced representation genomic datasets are increasingly becoming available from a variety of organisms. These datasets do not target specific genes, and so may contain sequences from parasites and other organisms present in the target tissue sample. In this paper, we demonstrate that (1) RADseq datasets can be used for exploratory analysis of tissue-specific metagenomes, and (2) tissue collections house complete metagenomic communities, which can be investigated and quantified by a variety of techniques.

**Methods:**

We present an exploratory method for mining metagenomic “bycatch” sequences from a range of host tissue types. We use a combination of the pyRAD assembly pipeline, NCBI’s blastn software, and custom R scripts to isolate metagenomic sequences from RADseq type datasets.

**Results:**

When we focus on sequences that align with existing references in NCBI’s GenBank, we find that between three and five percent of identifiable double-digest restriction site associated DNA (ddRAD) sequences from host tissue samples are from phyla to contain known blood parasites. In addition to tissue samples, we examine ddRAD sequences from metagenomic DNA extracted snake and lizard hind-gut samples. We find that the sequences recovered from these samples match with expected bacterial and eukaryotic gut microbiome phyla.

**Discussion:**

Our results suggest that (1) museum tissue banks originally collected for host DNA archiving are also preserving valuable parasite and microbiome communities, (2) that publicly available RADseq datasets may include metagenomic sequences that could be explored, and (3) that restriction site approaches are a useful exploratory technique to identify microbiome lineages that could be missed by primer-based approaches.

## Introduction

Next generation sequencing techniques have dramatically increased our understanding of the phylogenetic diversity of microbial communities, both in the environment and as metagenomic communities within multicellular hosts (Tremblay et al., 2015). Sequencing allows investigations of microbial communities without expensive, time-consuming, and sometimes unreliable culturing techniques (Browne et al., 2016). Metagenomic approaches also allow investigators to assess the relative abundances and activity levels of microbes as they occur in nature (Schloss et al., 2009; Kozich et al., 2013). New techniques for assessing microbial communities are continually being developed and refined. One area of concern for methods development is binding bias in primer sites that could result in some metagenomic taxa being overlooked in sequencing-based surveys (Clooney et al., 2016), if primer-based approaches are the first and only method of analysis for that community.

The most common approach to sequencing metagenomes requires researchers to amplify a pre-determined barcode primer that can bind to all target taxa. In this paper, we consider all mutualistic, commensal, and parasitic or pathogenic organisms to be part of the host’s metagenome. Relationships between hosts and the microbes and larger parasites that live in their tissues are often complex and context-dependent, so we prefer the most general term possible. One of the central problems in designing primers for metagenome-scale analysis is deciding which taxa should be considered during primer design (Dollive et al., 2013). User-friendly bioinformatics techniques and full mitochondrial and nuclear genomes of many taxa of interest have made it easier to design primers for metabarcoding techniques (Riaz et al., 2011). However, primers designed to fit known taxa in a community could completely miss unknown taxa that are present and potentially of interest to the investigator. Even within specifically-targeted higher taxa, primers often preferentially amplify some taxa and bind poorly to others, thereby potentially altering downstream community-scale analyses (Tremblay et al., 2015; Clooney et al., 2016).

Exploratory techniques that avoid primer-binding bias can help to identify target taxa for primer design, avoiding these problems in later barcoding analysis. Here, we demonstrate that enzyme-based reduced representation library approaches primarily used for host genomic analyses often co-amplify metagenomic DNA along with target host sequences. Our contribution is to provide a pipeline based on widely used analysis platforms, and present proof-of-concept examples on a range of sequence types. Double-digest restriction site associated DNA sequencing (ddRAD) approaches are relatively cheap compared to other non-primer-based next generation sequencing methods (Peterson et al., 2012). We present a protocol for identifying metagenomic DNA incidentally amplified during ddRAD and other short-read sequencing of multicellular host tissues. Previous work has shown that metagenomic sequences can appear in full genome assemblies, indicating that they may also be present in RADseq data (Orosz, 2015). We work across a range of host tissue types, including those commonly preserved as archival DNA in museum collections. We demonstrate that tissue samples collected primarily for genetic work on hosts can now be used to look at blood and tissue metagenome taxa, underlining the importance of long-term tissue preservation in publicly available collections. Additionally, our pipeline will allow researchers designing host-associated metabarcoding projects to survey publicly available datasets in order to refine their set of target taxa.

## Methods

### Sample collection and preservation

We sequenced tissues from multiple sources. We collected tail tissue samples (skin, muscle, and cartilage) in the field from two species of horned lizards, *Phrynosoma modestum* (six individuals) and *Phrynosoma cornutum* (nine individuals). We collected the samples using heat-sterilized scissors to avoid contamination, and stored them in RNALater in the field. These samples were collected in southwestern New Mexico in the summer of 2015 (permit number 3,606 to M. Grundler, University of Michigan IACUC protocol number PRO00006234). Samples were stored at ambient temperature in the field (for up to a month) and at −20 °C after being returned to the lab. We also sequenced DNA from night lizard (genus *Xantusia*) liver tissues (Natural History Museum of Los Angeles County, accession numbers TC1002, TC1003, TC1006, and RLB5221). Collection protocol is not available for the museum samples. The first three samples are *Xantusia vigilis* liver samples collected in 2012, and the last is a *Xantusia riversiana* liver collected in 1972. We also collected cloacal swabs (sterile rayon swabs, MW113) from two ribbon snakes, *Thamnophis sauritus*, and one water snake, *Nerodia sipedon*, in southeastern Michigan in the fall of 2015 (collected under a Michigan Scientific Collecting Permit 9-16-2015 to I. Holmes). We prevented the swab from coming into contact with the environment, the sampler, or the skin of the animals. We avoided contact with the skin around the cloaca by gently applying pressure to the ventral surface of the animal just anterior to the vent. This pressure slightly everted the cloaca, exposing the mucous membrane and allowing us to insert the swab cleanly. We removed the swab and placed it in a sterile 2 mL vial, then broke the shank so that the cap could be put on. We handled the shank of the swab only above the portion that will be preserved in the vial. The samples were transferred to −20 °C storage within hours of collection. We also sequenced samples from whole digestive tracts of two *Sceloporus jarrovi* preserved in 95% ethanol and stored at −80 °C (permit number SP673841 to Robert M. Cox). To acquire the samples, we dissected out the total lower intestines. We filled a sterile pipette tip with 100 μL of distilled water. We inserted the pipette tip into the intestine section, and depressed the plunger to force the water through the intestine. We collected the wash in a sterile 1.5 mL vial. The samples were sequenced in the fall of 2015.

### Laboratory protocol

We extracted total genomic and metagenomic DNA using Qiagen blood and tissue kits with a 12-hour incubation with proteinase-K prior to the spin column extraction. We used a double-digest RADSeq approach (Peterson et al., 2012), with the enzymes *Eco*R1 and *Msp*1 from New England Biolabs. We ligated barcoded Illumina adapters to the sticky ends left by the enzyme cuts, and used a PCR to attach barcoded Illumina primers to double barcode the sequence. We size-selected fragments with genomic inserts between 200 and 300 bp using a Pippin Prep cassette. We performed 125 bp paired-end sequencing the fragments on an Illumina HiSeq platform with V2500 reagents at the University of Michigan Sequencing Core.

### Publicly available sequence analysis

We downloaded three doubledigest RADseq datasets from NCBI’s Short Read Archive (SRR1947260 to SRR1947262). They are from the coral snake *Micrurus fulvius* (Streicher et al., 2016). Details of preservation are reported in the original paper. The authors report that samples were liver, heart, shed skin, or scales, and were preserved in ethanol or stored at −80 F. Details of storage on a per-sample basis were not available. Samples were restricted using the enzymes *Sbf*1 and *Sau*3A1, and paired-end sequenced on an Illumina HiSeq 2500 platform (Streicher et al., 2016).

### Sequence preparation

We demultiplexed the sequences using pyRAD, removed the low-quality sequences, and clustered reads within samples to 97% identity (pyRAD steps 1–3) (Eaton, 2014). We chose this clustering threshold because many microbial ecologists use a 3% difference in sequences to identify operational taxonomic units. We used the resulting fasta file of clustered sequences for each individual (the pyRAD *.edit file) for all further analyses. Any combination of sequence quality control and clustering programs can be used for this step, for example FastQC or Trimmomatic for filtering (Andrews, 2010; Bolger, Lohse & Usadel, 2014), or vsearch for clustering (Rognes et al., 2016). For the *Phrynosoma* and *Xantusia* samples, we continued the pyRAD pipeline to cluster reads across individuals, and used the resulting “*.loci” file for further analyses. In the pyRAD *.loci files, the sequences for each locus are listed in a group, with the individual that provided the sequence identified in the name of that sequence. A standard line break string separates the sequences for each locus. We used a custom R script to take the first sequence for each locus and combine them into a fasta file to be passed to our analysis pipeline (Appendix 1).

### Investigating metagenomic sequences

We use NCBI’s discontinuous megablast algorithm to compare all sequences from the *.edit and *.loci files to reference sequences in the online NCBI nucleotide database (Camacho et al., 2009). We use the R package taxize to find the genus and species of each sequence (Chamberlain & Szocs, 2013; Chamberlain et al., 2016). We discard results that aligned to more than one kingdom or phylum with greater than 80% identity. To assess how the threshold for similarity affected the number of sequences that can be identified to phylum, we imposed percent similarity thresholds to the closest matching sequence of 70%, 80%, 85%, 90%, 95%, and 97%. To assess the distribution of parasite sequences across hosts, we screened sequences that clustered across individuals of two horned lizard species, *P. cornutum* and *P. modestum*. We performed a similar analysis on samples from two *Xantusia* species. Finally, we built rarefaction curves using the R package vegan to identify the depth of sampling necessary to identify all genera of metagenomic DNA present in the sample (Oksanen et al., 2018). For each genus for which we have tissue metagenomes (*Phrynosoma*, *Xantusia*, and *Micrurus*) we created a community matrix in which samples are rows and columns are the the number of Chordata sequences, and the number of sequences in each genus of blood parasite. We set a 90% identity match for this analysis.

## Results

**Figure 1:**
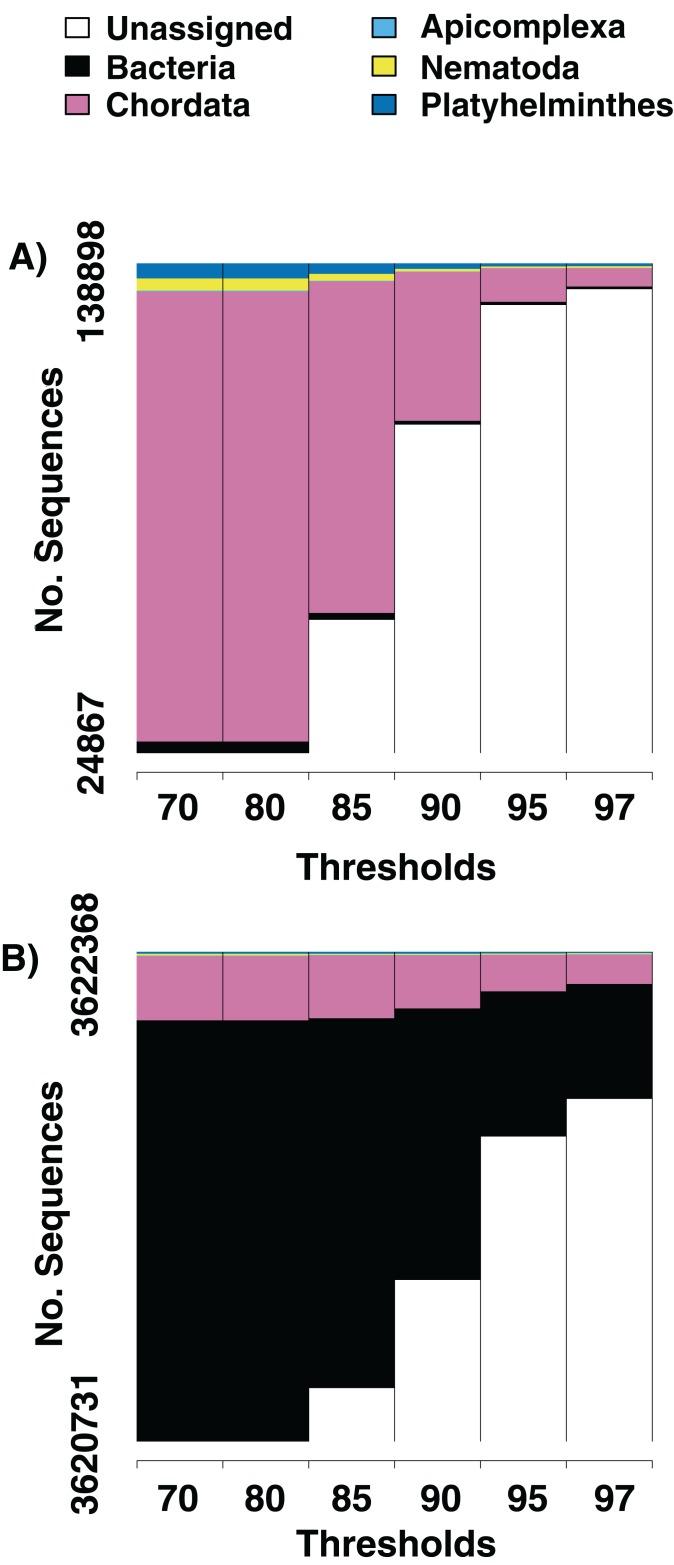
Numbers of parasite bycatch sequences at increasing thresholds of similarity to reference sequences. Changes in the relative number of sequences that aligned to an identifiable phylum in the NCBI database at six thresholds for percent similarity between the sequence and its closest GenBank hit. (A) Is from Texas horned lizard, *Phrynosoma cornutum*, tail tissue, (B) is from a ribbon snake, *Thamnophis sauritius*, cloacal swab.

We identify sequences that match with reference flat worms (Platyhelminthes), round worms (Nematoda), and Apicomplexans (the phylum that contains malarial parasites) from the majority of tissue samples we sequenced (Table 1). When sequences are examined at a 97% similarity to reference sequence threshold, we find that from an average of 1,252,549 (s. e. +/− 1,080,872) sequences per sample, 466 (s. e. +/− 301) are identifiable host sequences, 40 (s. e. +/− 70) are from platyhelminths, and 20 (s. e. +/− 34) are from nematodes. On average, we identify 3.2 (s. e. +/− 1.7) unique playthelminth taxa and 2.9 (s. e. +/− 1.8) unique nematode taxa per individual. The large majority of sequences do not have any significant match in the BLAST database at the 97% similarity threshold (Table 1). We present two examples in which we alter the threshold for similarity of a sequence to its top hit in GenBank from 70%, 80%, 85%, 90%, 95% to 97% (Fig. 1). Increasing the similarity threshold causes the number of sequences matched to each phylum decrease, but generally not to go to zero. Results from gut samples show more and greater diversity of metagenomic taxa relative to sequences from muscle tissue.

**Table 1:** Sequences aligned with 97% similarity to host or parasite templates.

Sample	Total	Chordata	Platyhelminthes	Nematoda	Apicomplexa	Species	Sample type
**corn1**	379,196	12	1	0	0	*Phrynosoma cornutum*	Tail tissue
**corn2**	138,897	533	61	49	0	*Phrynosoma cornutum*	Tail tissue
**corn3**	846,104	564	29	5	0	*Phrynosoma cornutum*	Tail tissue
**corn4**	1,918,882	292	9	1	0	*Phrynosoma cornutum*	Tail tissue
**corn5**	2,591,254	501	10	1	0	*Phrynosoma cornutum*	Tail tissue
**corn6**	550,893	543	31	7	0	*Phrynosoma cornutum*	Tail tissue
**corn7**	57,273	633	313	138	0	*Phrynosoma cornutum*	Tail tissue
**corn8**	132,434	10	0	3	0	*Phrynosoma cornutum*	Tail tissue
**corn9**	927,740	213	13	8	0	*Phrynosoma cornutum*	Tail tissue
**mod1**	2,052,740	603	23	4	0	*Phyrnosoma modestum*	Tail tissue
**mod2**	2,423,509	1,363	46	11	0	*Phyrnosoma modestum*	Tail tissue
**mod4**	3,070,606	564	12	4	0	*Phyrnosoma modestum*	Tail tissue
**mod5**	1,018,036	622	28	11	0	*Phyrnosoma modestum*	Tail tissue
**mod6**	298,447	554	28	22	0	*Phyrnosoma modestum*	Tail tissue
**mod7**	111,733	10	1	0	0	*Phyrnosoma modestum*	Tail tissue
**RLB5221**	342,173	478	9	12	0	*Xantusia riversiana*	Liver tissue
**TC1002**	91,094	759	212	231	1	*Xantusia vigilis*	Liver tissue
**TC1003**	2,154,606	6	1	0	0	*Xantusia vigilis*	Liver tissue
**TC1006**	836,745	1,360	73	30	0	*Xantusia vigilis*	Liver tissue
**SRR1947260**	350,330	2,966	1	3	0	*Micrurus fulvius*	Streicher et al. (2016)
**SRR1947261**	296,572	7,343	5	2	0	*Micrurus fulvius*	Streicher et al. (2016)
**SRR1947262**	323,453	694	3	2	0	*Micrurus fulvius*	Streicher et al. (2016)
**Tsauritus1**	260,079	1,148	4	3	2	*Thamnophis sauritus*	Cloacal swab
**Tsauritus2**	1,174,345	3,594	9	11	2	*Thamnophis sauritus*	Cloacal swab
**Nsipedon**	1,772,451	6,762	9	22	2	*Nerodia sipedon*	Cloacal swab
**Sc0055**	3,622,367	98	2	4	1	*Sceloporus jarrovi*	Dissected gut
**Sc0100**	2,715,018	577	8	9	0	*Sceloporus jarrovi*	Dissected gut

**Note:**

Preserved host muscle tissue also preserves genetic material from three major taxa of parasites, Platyhelminthes, Nematoda, and Apicomplexa. Only sequences that had 97% or greater similarity to a sequence in GenBank are included. Total number of sequences for each sample included for reference.

**Figure 2:**
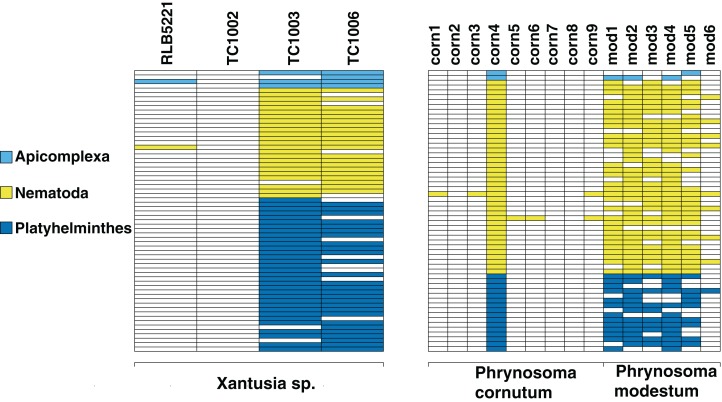
Identifiable metagenomic sequences between congeneric hosts. Data from each host is shown in columns; rows indicate distinct, identifiable metagenomic sequences. White sequences are absent from a given host, sequences with color were detected in a given host. For comparison purposes, we assign different colors to each parasite phylum.

We screen a dataset of three desert night lizards (*X. vigilis*) and one island night lizard (*X. riversiana*). Nineteen unique metagenomic sequences have two or more representatives in the final assembly (Fig. 2), out of 81,966 total sequences. One hundred and ninety-seven of the sequences in that assembly align with Chordata reference sequences with 97% similarity. Fifteen sequences out of the final assembly align to Platyhelminthes sequences with 97% similarity. Fourteen of these match with *Protopolystoma xenopodis* and one with *Diphyllobothrium latum*. Four nematode sequences align with the species *Nippostrongylus brasiliensis*, *Strongyloides stercoralis*, *Soboliphyme baturini*, and *Elaeophora elaphi* at 97% similarity.

**Figure 3:**
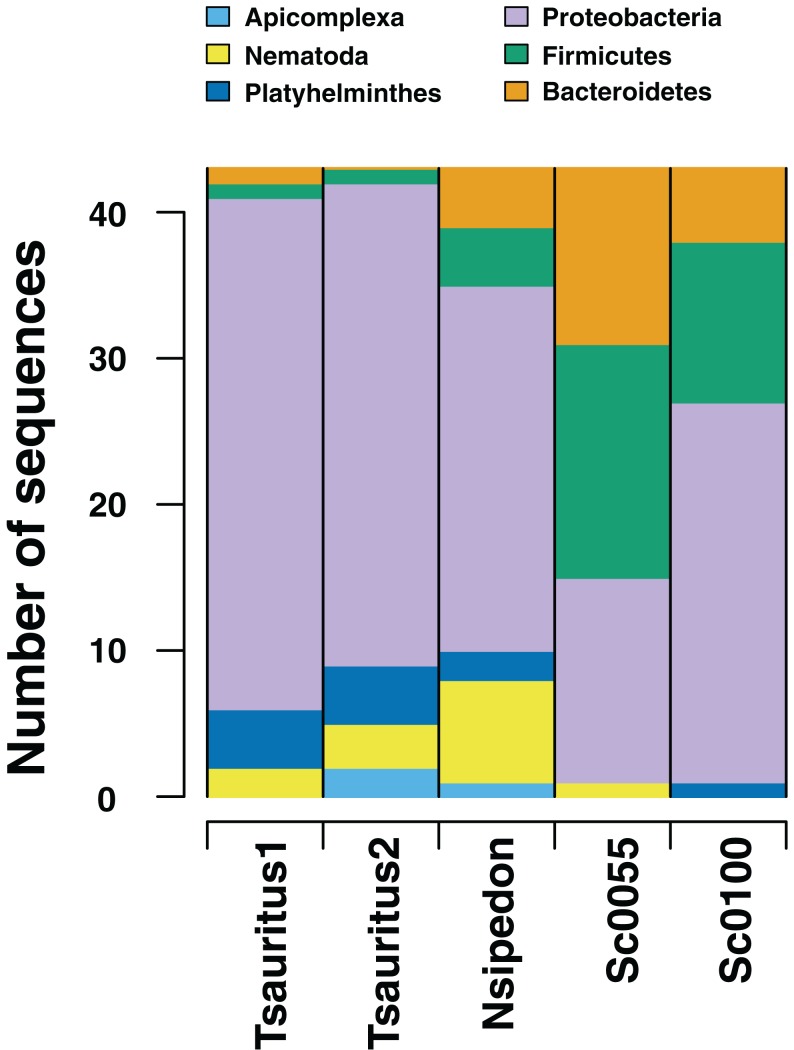
Numbers of gut microbiome phyla from two collections techniques. RADseq approaches amplify a range of identifiable bacterial and eukaryotic sequences from intestinal rinse (*Sceloporus jarrovi*) and cloacal swab (*Thamnophis sauritus* and *Nerodia sipedon*) sequences.

We also investigate the efficacy of the ddRAD approach in surveying the diversity of the hindgut microbiome. We find that the approach reliably returns sequences from the three most common phyla of gut bacteria in reptiles (Fig. 3): Proteobacteria, Firmicutes, and Bacteroidetes (Colston & Jackson, 2016). We also retrieve sequences from Platyhelminthes, Nematoda, and Apicomplexa. All three phyla are known gut community members (De Chambrier & De Chambrier, 2010; Molnár et al., 2012; Peichoto et al., 2016). In addition to the phyla common to all four samples, we find taxa specific to individual hosts. These include the bacterial phyla Actinobacteria, Chloroflexi, Tenericutes, Planctomycetes, Cyanobacteria, Synergistetes, Deinococcus-Thermus, Armatimonadetes, Thermotogae, Verrucomicrobia, Ignavibacteriae, Spirochaetes, Fibrobacteres, Acidobacteria, Fusobacteria, and the Archaea phylum Euryarchaeota. We also find the fungal taxa Ascomycota, Basidiomycota, and Entomophthoromycota. The first two samples also contain sequences that align with Cnidaria. These are likely Myxozoans, a branch of cnidarians that parasitize vertebrate guts (Foox & Siddall, 2015).

**Figure 4:**
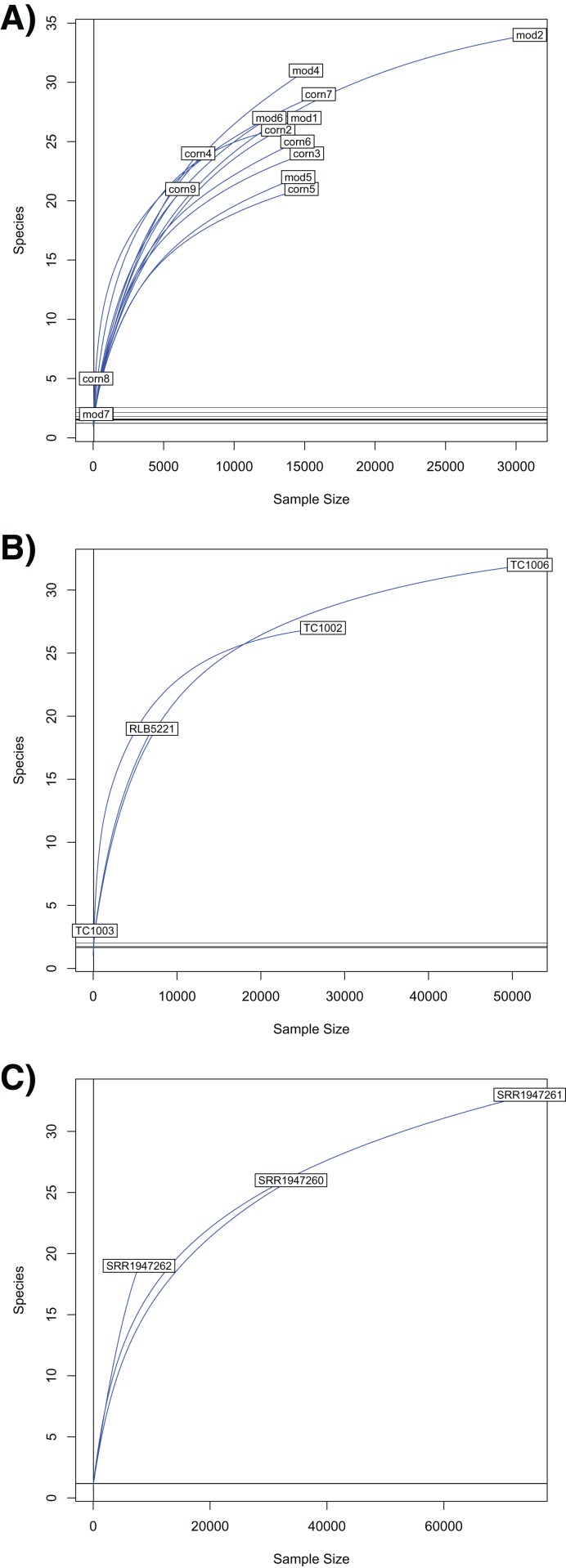
Rarefaction curves for Chordata and blood parasite metagenomic sequences. Rarefaction curves for genera of blood parasites and host sequences from the three genera of hosts: *Phrynosoma* (A), *Xantusia* (B), and *Micrurus* (C).

Our rarefaction curves show that most tissue datasets need to have at least 40,000 identified sequences to capture metagenomic communities (Fig. 4). Some of our samples (notably corn8, mod7, and TC1003) fall far short of that threshold, while others are closer to it but still likely to have undetected metagenomic information. Raw data files use in this paper are available at https://doi.org/10.6084/m9.figshare.5593522.v1.

## Discussion

We find that metagenomic sequences can be identified from a range of tissue types with variable preservation histories (Table 1). Our screened tissues included liver preserved in ethanol from a museum collection, lizard tail-tip tissues preserved in RNALater, lizard guts preserved in ethanol, and cloacal swabs preserved in ethanol. All preservation and sample types yield metagenomic sequences, indicating that most or all of the tissues currently preserved for DNA extraction in museum collections worldwide (Yeates, Zwick & Mikheyev, 2016) are also repositories of metagenomic information. These repositories of metagenomic sequences can be analyzed using a range of approaches, including the RADseq exploratory techniques we present here and more conventional amplicon-based metagenomic profiling. Our exploratory approach shows that the majority of sequences generated by RADseq for our host tissues are not identifiably similar to any publicly available reference sequence. Less than 1% of the sequences that cluster across individual hosts are 97% similar to any NCBI GenBank sequence. Of those that do hit our similarity threshold, the vast majority are Chordate (host) sequences, when tissue samples are the source of DNA. Other DNA sources, such as cloacal swabs and intestinal rinses, have different taxonomic profiles. However, a number of sequences amplified from lizard tail and liver tissue align with phyla known to occur in the blood and tissue metagenome: Platyhelminthes (blood flukes and relatives), Nematoda (round worms), and Apicomplexa (malaria parasites and relatives). More that 50% of the metagenomic sequences identified in *Phrynosoma* were found in both species present at the site, indicating that the parasites are common within and between closely related host species.

The double digest RADseq approach worked across a range of sample types, including standard tissue samples commonly used for host genetic analysis (liver and muscle), and less conventional sources, such as cloacal swabs and rinses from preserved digestive tract. Any metagenomic source that can produce the necessary 100–200 ng of DNA for the ddRAD protocol should be tractable for this type of analysis (Peterson et al., 2012). All tissue types produced large numbers of sequences that could not be matched to publicly available references at any similarity threshold (Fig. 1). As percent identity threshold levels increased, the number of sequences that matched to the host dropped quickly. There was no threshold that completely excluded sequences that matched common parasite or microbiome phyla, indicating that they were present in the extracted DNA with high confidence.

### Limitations and caveats

The major limitation of our approach is that it relies on public databases to determine the taxonomic identity of sequences. However, public databases do not accurately reflect the diversity of metagenomic taxa. For example, we recover relatively few Archaea sequences from our hind-gut samples. We hypothesize that this reflects the relative lack of Archaea genetic sequences in GenBank to compare against, rather than an absence of Archaea from our samples. However, the number of publicly available, taxonomically identified reference sequences is quickly increasing, so this source of bias should be reduced in the future. Second, our identifications are based on randomly restricted DNA samples, rather than widely-accepted barcode sequences. These sequences can’t be corrected for copy number variation, and we have little to no ability to determine whether two different sequences represent different individuals or whether they are two separate samples of the genome of a single individual. Due to the inherent stochasticity of the ddRAD approach, this method should not be used to quantify the relative or absolute abundance of metagenomic communities. Finally, the sequences in this paper are from an Illumina HiSeq platform. Negative controls are not recommended on this platform, as running one leads to uneven ratios in barcode sequences, which can damage the sequencing quality for the entire run. The lack of negative controls is one reason that this approach should be considered exploratory, rather than as a method for quantifying microbial load in specimens. Well-designed primer sets can account for all of these problems, and should be used to answer questions about relative abundances of metagenomic lineages or community structure.

We note some unexpected taxonomic identifications in our sequences. Specifically, we note that some of the sequences align with highest percent identity with arthropod sequences, or with Streptophyta. These sequences may represent sequencing error that alters a highly conserved host or metagenomic sequence to erroneously align more closely with a non-target sequence. Alternatively, sequences could be labelled incorrectly in the NCBI database, or they could be contamination in genome assemblies (Orosz, 2015). Regardless of the source, this taxonomic error indicates that our method should be used for exploratory purposes only.

### Ecology of metagenomic sequences

All of the putative parasite taxa that we can identify with 97% certainty are known parasites of vertebrate hosts. *Protopolystoma xenopodis* is known from African clawed frogs, in which it attaches to the kidney and feeds on blood, thereby potentially releasing its own DNA into the host’s bloodstream (Theunissen, Tiedt & Du Preez, 2014). *Diphyllobothrium latum* is known from the digestive tracts of a range of vertebrates, including mammals and fish (Wicht et al., 2009; Schurer et al., 2016). *Nippostrongylus brasilensis* and *Strongyloides stercoralis* are nematodes known from mammals. Their lifecycle begins with free-living juveniles that find a host and bore into the bloodstream through the skin. The juveniles migrate to the lungs, where they develop into adults before entering the digestive tract to breed (Koutz & Groves, 1953; Haley, 1961). *Soboliphyme baturini * is known from mammals, and infects the stomach (Zarnke et al., 2004). *Elaeophora elaphi* occurs in red deer, where it lives in the portal vein near the heart (Carrasco et al., 1995). While the sequences we detected are probably not the same species as their closest match, they should be closely related, and are likely to have similar life histories. All of the life histories here indicate that parasite DNA could plausibly be shed into the bloodstream.

We found many families of bacteria that are known from vertebrate guts, and some that have not previously been recorded. Actinobacteria, Acidobacteria, Cyanobacteria, Fusobacteria, Spirochaetes, Synergistetes, and Tenericutes have been reported from wild snake hindguts (Colston, Noonan & Jackson, 2015). Plancomycetes and Verrucomicrobia have been found in the guts of wild apes (Yildirim et al., 2010). Euryarchaeota, Deinococcus-Therums, Thermatogae, and Fibrobacteres have been found in dog gut microbiomes (Swanson et al., 2011). Chloroflexi has been recorded from human guts (Campbell et al., 2014), and Synergistetes has been recorded from the gut of young calves (Li et al., 2012). To the best of our knowledge, the bacterial phyla Ignavibacteriae and Armatimonadetes, and the fungal phylum Entomophthoromycota have not previously been reported from vertebrate hind microbiomes. A species in Entomophthoromycota has been found in a cyst in the esophagus of a rat snake, *Elaphe obsoleta* (Dwyer et al., 2006). Other Entomophthoromycota are pathogens of invertebrates (Gryganskyi et al., 2012), indicating that they are capable of invading multicellular hosts. Ignavibacteriae is a sister phylum to Bacteroidetes and Chlorobi, both known from gut microbiomes (Podosokorskaya et al., 2013). The phylum has been sequenced from wastewater, indicating that it can survive in organic waste (Meng et al., 2015). Armatimonadetes is primarily a soil phylum, but also participates in plant rhizobial communities (Tanaka et al., 2012), and has been found in mosquito salivary glands (Sharma et al., 2014) and decomposing swine manure (Tuan et al., 2014). The three new phyla are all reasonable candidates for the gut microbiome, as they are known to occur within multicellular host tissues. However, caution should be exercised because both the known and novel taxa we identified from the gut samples can also be found in environmental samples. Further study, using carefully selected barcode loci, should be undertaken before these taxa are considered an established part of the gut microbiome.

## Conclusion

Our results demonstrate the value of long-term storage of a variety of tissue types in publicly available collections. Techniques that have not yet been developed at the time of tissue collection may later become available, rendering the samples and their metadata (geographic locality, time of year collected, and other ecological data) highly relevant. Similarly, current publicly available short read datasets may include as yet unrecognized metagenomic sequences. Investigators designing amplicon-based approaches to microbial communities in specific host tissue types could mine available datasets to gain an understanding of the taxa they should be targeting.

## Supplemental Information

10.7717/peerj.4662/supp-1 Supplemental Information 1 R code for pipeline. In combination with the pyRAD and blastn programs, this code will allow users to find metagenomic sequences in RADseq and similar datasets.

## Additional Information and Declarations

### Field Study Permissions

The following information was supplied relating to field study approvals (i.e., approving body and any reference numbers): Field experiments were approved by New Mexico Game and Fish, Arizona Game and Fish, and Michigan Fish and Game.

### Data Availability

The following information was supplied regarding data availability: Holmes, Iris (2017): files for ‘Natural History Bycatch’. figshare. Fileset. Figshare: https://doi.org/10.6084/m9.figshare.5593522.v1.
